# Assessment of Risk Factors in Patients With Myocardial Infarction

**DOI:** 10.5539/gjhs.v8n1p255

**Published:** 2015-05-28

**Authors:** Fatemeh Kiani, Nasrin Hesabi, Azizollah Arbabisarjou

**Affiliations:** 1 Health Promotion Research Center, Zahedan University of Medical Sciences Zahedan, IR, Iran

**Keywords:** risk factors, myocardial infarction

## Abstract

**Background:**

Coronary artery diseases (CAD) are one of the important health problems in the world, although considerable progresses have been made to decrease the mortality, they are still the first cause of death in many countries. Hence, the necessity of examining effective factors and risk factors on CAD can be one of the most important health priorities in many countries like Iran.

**Objective:**

This study was performed to assess the risk factors in patients with myocardial infarction (MI) in Zahedan.

**Materials and Methods:**

This is a cross sectional study in which 213 patients were examined. They had been diagnosed to have heart failure. Data gathering took 18 months. Data gathering tool was a designed checklist which was filled up by an experienced nurse during interview. Obtained results were recorded in files and analyzed in SPSS 21.

**Results:**

Results showed that 70% of patients were women and only 30% were men. 48% of them were illiterate and patients mean age was 58.3. SD had been 12.6. The mean of pain onset time till referring to hospital was 11 hours with SD of 2.1. 17% of patients (coronary artery diseases history), 25.5% (hypertension history), 26% (diabetes history), 15.5% (cholesterol history), 13% (smoking) and 3% have reported CABG history. The majority of people who referred had inferior MI (40.4%). 67.1% normal rhythm, 2.8% atrial fibrillation and 16% had ventricular tachycardia. Statistical tests showed a significant correlation between sex and the mean of referring time (p<0.05) but the relation between age and referring time was not significant.

**Conclusion:**

Effective risk factors on MI were recognized in this study. Some of them such as age, sex and education cannot be modified but many are controllable such as hypertension, diabetes, cholesterol, and smoking and on time referring after pain onset. Having considered the results of this study health promotion for society and especially vulnerable people can be provided by omitting or reducing risk factors.

## 1. Background

Coronary heart disease (CHD) is the most common serious disease in industrialized communities and a fast developing health problem in developing countries. These diseases have caused mortality in developed countries more than other diseases and impose numerous social and economic costs. These diseases are now seen in countries with low or average income which also have the majority of population. These diseases will probably turn into the most common cause of death in world till 2020 (Fauci et al., 2008).

During recent decades developed countries have been able to decline the coronary heart diseases mortalities significantly by preventive actions. They have passed the outbreak stage of this epidemic and the disease mortality has dramatically declined in them (Rossignol et al., 2012).

It seems that the mortality of these diseases will increase in developing countries due to lack of familiarity with coronary heart diseases risk factors and failure to comply preventive principles. Based on health ministry statistics mortality of these diseases is progressing in Iran so that 70 per cent of death in Iran is due to heart diseases and also 15 to 17 million in the world annually (Akbari, Mohammadzadeh, Rajabpoor, & Azim Poor, 2009).

One of the diagnosis methods is examining the affected patients (Sezavar, Valizadeh, Moradi Lakeh, & Rahbar, 2010). Some of the risk factors of coronary heart disease are uncontrollable like senility, being male and history of atherosclerosis that are considered uncontrollable as risk factors but many of them can be modified like hypertension, hyperlipidemia, mellitus diabetes and smoking cigarette which are commutable risk factors of coronary artery disease (Andreoli, Carpenter, Griggs, & Benjamin, 2007). Studies results shows that CAD is not incidental and affected people can be found by clinical symptoms (Kwak, Myung, Lee, & Seo, 2012). Cigarette is the most preventive risk factor. The effect of smoking cessation in smokers with coronary artery stenosis equals to that of surgery (Sivaraman, Hausenloy, Wynne, & Yellon, 2010). The relationship between nutritious factors and blood lipids levels has also been examined in human groups in randomized studies (Ellingsen, Hjerkinn, & Arnesen, 2006; Baxter, Coyne, & McClintock, 2006). Now even the positive effect of cholesterol decline in healthy people who have normal cholesterol level has been proved (Sivaraman et al., 2010).

Clinically cigarette decreases high density lipoproteins (useful lipids) and increases low density lipoproteins (harmful lipids) and blood glucose. Reduce in serum cholesterol average and hypertension after CAD has been proved (Kathleen, Zubair, & Belgin, 2006; Yarnell, Christopher, & Hugh, 2005). Clinically cardiac enzymes and ECG can be one of ways of diagnosing heart failure. Cardiac enzymes changes can be observable in ECG of myocardial infarction patients (Zipes, Bonow, & Braunwalds, 2006).

Prevalence of cardiovascular diseases is high in our society. Preventive measurements can be done by precise recognition of patients. Other studies have only mentioned specific aspect of disease. The purpose of the study was to assess risk factors in MI patients and determine clinical criteria for patients with MI who referred to Khatm-Al Anbia-hospital affiliated to Zahedan University of Medical Sciences, Iran during 2013-2014. This hospital is the oldest hospital in Zahedan and one of the largest hospitals that have CCU wards. It is possible to provide more accurate information for health authorities by determining risk factors.

## 2. Materials and Methods

This cross-sectional study was done using interview with MI patients and their records (based on WHO/AHA criteria). Diagnosing severe MI requires the followings based on WHO/AHA criterions.

1. Increase or decrease of biochemical typical markers of myocardial necrosis with at least one of the following: signs of ischemia, pathologic Q wave rise in ECG changes in ECG that is indicative of ischemia (rise or fall of ST segment).
2. Pathologic factors of one AMI (Libby, Bonow, Mann, & Zipes, 2008).

The study was done through interviews and patients’ records. 213 patients with acute MI who referred to Khatam Al Anbia hospital in Zahedan filled out checklists during 18 months. Patients with definitive diagnosis of MI were hospitalized and examined in CCU wards of Khatam hospital. An experienced and trained nurse gathered the data.

The used checklist was consisted of four parts. The first part included demographic data like age, sex, education and residence. The second part included pain onset time, emergency hospitalization time, CCU hospitalization time. The third part included questions related to coronary artery disease history and its duration, hypertension history and its duration, diabetes history and its duration, cholesterol and its duration, history of smoking cigarette and its duration, CABG history and its duration, receiving thromboembolism kinase drug, laboratory tests such as Troponin T,I; CKMB; rhythm type, hospital discharge status and drugs taken (anticoagulant, nitrite, Clonidine, Statins, diuretics and beta-blockers) finally patients information and kinds of MI have been completed. Kinds of MI, the most common observed dysrhythmia, and drugs taken have been mentioned in the fourth part.

The data were entered to the SPSS21. Significance level index was 0.05.

## 3. Results

There were 70% (149) male participants and 30% (64) females. The mean of participants age 58.3, SD 12.62, minimum age 29 and maximum age was 100. 48.4% of participants were illiterate. 24.9% had elementary education, 16.4% diploma, and 26.7% university degree. 10.3% of those with MI had university degree. 88.3% of participants resided in Zahedan and others resided in Sistan and Balouchestan province towns or out of province. Pain onset time had been 14% (in most cases) between 8 and 9 o’clock, and totally 33.5 % of patients have stated pain onset time between 9 and 12 in the noon. 36.6% of them have reported the pain between 13 till 20 and 25.9% between 21 and 6 o’clock.

The mean of pain onset time duration until emergency reception was 11 hours with SD of 21. Referring time was at least 15 minutes and at most 169 hours. The time interval between emergency receptions until CCU had been at least 10 minutes and at most 2 days.

Frequency of other diseases history of patients are the following:

22% history coronary artery disease, 25.5% history of hypertension, 26% history of diabetes, 15.5% cholesterol history, 13% history of smoking cigarette, and 3% have reported CABG history (Table 1).

**Table 1:** Assessment of risk factors in patients with MI

Risk factor	Number	percent
CAD history	36	17
Diabetes	56	26
Hypertention	54	25.5
CABG	7	3
Cholesterol	32	15.5
Smoking cigarette	28	13
Total	213	100

**Different kinds of reported MI**

The findings showed different kinds of MI as following:

Inferior lateral MI (4.7%), inferior MI (40.4%), anterior MI 25.5%, anteseptal MI 9.4% and 20% had been other MIs (Table 2). Regarding ST segment changes, ST segment elevation was observed in most cases (90%).

**Table 2:** Different kinds of reported MI in Patients

Kind of MI	Number	Percent
Inferior lateral	10	4.7
inferior	85	40
Anterior	55	25.5
Ant septal	20	9.4
Other MI	43	20
Total	213	100

A statistically significant correlation was observed between pain onset time until referring to hospital with sex so that the mean of referring time was 9 hours for men and 17 hours for women. 67.1% of Patients had normal rhythm. 2.8% atrial fibrillation, 16% ventricular tachycardia, 3.1% right and left bundle branch block, 2.8% Mobitz type 2, 1% CHB and 0.5% had junctional rhythms.

The mean of participants’ age was 58.3+-12.6. (Minimum 29 and maximum 100). This study is very similar to that of Sezavar & et al; they have reported the mean of age of patients with MI 59 which is more than that of south Asia and Middle-East patients (14). Youngest population of patients with the first AMI resided in south Asia (age mean 53 years old) and Middle East (51 years old). The oldest population had been residents of Western Europe, China and Hong Kong (63 years old).

70% of participants were men in our study which has been mentioned 84% versus 16% in other studies, this ratio had been more for men than women in other studies for example these have been proved in studies of Yousef et al. (2004) and Ismail et al. (2004). 20% of all MI receptions have been MI less than 55 for men and less than 65 for women in U.K (Chow, Pell, Walker, O’Dowd, Dominiczak, & Pell, 2007).

Regarding ST segment changes, ST segment elevation was observed in most cases (90%). A statistically significant correlation was observed between pain onset time until referring to hospital with sex so that the mean of referring time was nine hours for men and 17 hours for women. Patients (67.1%) had normal rhythm. atrial fibrillation(2.8%), ventricular tachycardia (16%), right and left bundle branch block (3.1%), Mobitz type 2 (2.8%), CHB (1%) and 0.5% had junctional rhythms.

The mean of participants’ age was 58.3+-12.6. (Minimum 29 and maximum 100). This study is very similar to that of Sezavar & et al. In their study, they have reported the mean of patients age with MI 59 which is more than that of south Asia and Middle East patients (Yusuf et al., 2004). Youngest population of patients with the first AMI resided in south Asia(age mean 53 years old) and Middle East (51 years old). The oldest population had been residents of Western Europe, China and Hong Kong (63 years old). Participants (70%) were men in our study which has been mentioned 84% versus 16% in other studies. This ratio had been more for men than women in other studies for example these have been proved in study of Esmaeel and Yousef (Fred, 2010; Ismail et al., 2004; Yusuf et al., 2004). 20% of all MI receptions have been in England; MI less than 55 for men and less than 65 for women (Chow et al., 2007; Ismail et al., 2004).

Clinical assessment sciences institute in Canada carried out a study on 4403 patients in Ontario Canada. They all had heart attack records which showed that mean of their age (67.3 years) and 33.7% were women. Statistics showed that numbers of married men who have been taken to hospital after heart attack faster were more than single ones, but in contrast there has been no relationship between marital statuses of women with arrival speed to hospital after experiencing chest pain related to heart attack which indicates that women take care of their husbands better (Chow et al., 2007; “Iranian Students’ News Agency - ISNA,” 1998-2013). 48.4% of participants were illiterate in the present study. Results of a study showed that the majority of patients 79.1% did not have any information about the initial symptoms of MI (Akbari et al., 2009). Illiteracy lead to ignorance and uneducated people certainly have lower level of health behaviors and preventive behaviors. Providing information by mass media and correct planning by health centers is needed for secondary prevention. It is recommended to provide necessary information for public education and also training and counseling for older patients with higher risk of heart disease.

23.5% of patients have reported the pain onset time between 12:00 till 6:00 in the morning. Results of the study of Goff et al. (2007) showed that analysis of patients with MI diagnosis indicated that circadian cycle for MI had been between 6:00 am and 12:00 pm (Ellingsen et al., 2005). Most MI occurrence time had been in the morning, preventing it needs planning for public awareness in societies which are more prone to acute MI. The mean of pain onset time duration until emergency reception is eleven hours. Another study which was carried out in Uremia. It was mentioned 80 hours and 36 minutes (Akbari et al., 2009). Streptokinase drug which is recommended as a fibrinolytic drug for patients with chest pain, its highest effect is the first 30 minutes after MI (‘Iranian Students’ News Agency - ISNA,” 1998-2013; Janszky et al., 2012; Khan et al., 2007). Other resources have mentioned that drug effectiveness rate for revascularization (by tromboliza, angioplasty or both) depends on the interval of coronary artery obstruction beginning and reperfusion (Herman & Walsh, 2011; Janszky et al., 2012; Taghadosi, Seyedi, & Mosavi, 2007).pain onset time had been 11 hours in our study while results of Mirzaei’s study which was carried out in Kerman showed that it lasts 2 hours from chest pain beginning till referring to health centers that is much different from our study (Bagherian Sararoodi, Saneei, & Bahrami Ehsan, 2010; Mirzaei, Mohammad, & Bagherian, 2005).what reasons have caused this delay? It seems that we can divide delay time from beginning till hospitalization at CCU ward in the following three stages:

(1). Time needed for patients to make decisions, state their illness and ask help.
(2). Moving the patient from the place of pain onset to emergency or health centers.
(3). Delay time between hospital emergency to hospitalization at CCU ward, but in these all three stages other factors are also important.

The second part has not been attended in our study; in fact the first and the third stages are combined in our study. It was recognized in this study that most of the delay time had been related to patient decision making for asking help and other stages were less important regarding wasting time. Results of the study of Taghadosi et al. (examining the reasons and amount of MI patients referring delay to Shahid Beheshti hospital of Kashan in 1383 showed that 89% of patients had more than 8 hours delay in referring to emergency (Anand et al., 2008; Taghadosi et al., 2007). Lokker has stated pain beginning time till reaching to hospital 110 minutes and African women had the most delay (Lokker et al., 2015). Women had more delay than men in our study. The reason of this delay may be higher pain tolerance threshold in women or more MI prevalence in men. Women do not attribute chest pain to heart and its diseases and do not do anything to reduce it (Afzal, Korniyenko, & Haq, 2015). Women suffer from MI when they are older which may decrease pain for them and make them more tolerable (Taghadosi et al., 2007). 30.5% of patients had hypertension history in this study. The role of hypertension in heart complications and its outcomes in MI patients is not unknown to anyone. Probably patients after MI with hypertension expect more MI unpleasant outcomes and consider their illness less controllable and curable compared to patients without hypertension (Bagherian Sararoodi et al., 2010; Gaziano, 2005). Hypertension risk factors and diabetes had more relation to MI in women than men in study of Anand et al. (Anand et al., 2008; Kazemy & Sharifzadeh, 2010). Tobacco smoking has been under the influence of cultural and historical backgrounds of societies; women smoked less than men in most societies which clearly explains low MI rate in young women compared to men. Molarris et al. recognized in their study that among attributed risk factors to MI patients, smoking cigarette and hypercholesterolemia is more in young individuals and hypertension, diabetes and CAD is more in older ones (Morillas et al., 2002). Studies have demonstared that heavy smoking is the most important factor of early MI (Gaziano, 2005). Our study does not conform to other studies in this background. Diabetes 26%, hypertension 25.5%, CAD history 17%, high cholesterol history 15.5% CABG 3% and smoking 13% had been the most important risk factors in the aforementioned patients respectively.

It was recognized in other studies that annual mortality had been 26% in diabetic patients and 14% in non-diabetic ones. The mortality of diabetic patients with MI had also been higher than non-diabetic ones (Akbari et al., 2009; Erlinge et al., 2014; Kazemy & Sharifzadeh, 2010; Lee & Chou, 2003). This ratio rises if hypertension and diabetes are both existent (Kazemy & Sharifzadeh, 2010). Anatomically the most prevalent kind of MI is respectively inferior 40.4%, anterior 25.8%, anteseptal 9.4% and the least is lateral MI. It is 53% inferior and 40% anterior in Soltani et al. study (Kazemy & Sharifzadeh, 2010; Soltani MH, 2005). Infarction section is a prognosis factor and anterior infraction has a more severe prognosis (Perkins-Porras, Whitehead, Strike, & Steptoe, 2009). It was recognized in Soltani’s study that patients with wide anterior infarction have had more intra hospital and annual death compared to the other infractions. Anterior infarction causes 31% of death compared to the 15% of inferior infraction (Soltani MH, 2005; Z, 2004).

It was recognized in our study that 90% of patients show ST segment rise while other resources have emphasized that early thrombolytic treatment reduces mortality especially that of MI with rise of ST segment and lethal rhythms. Since thrombolytic treatment depends on time, treatment beginning less than one hour is important for these patients (Banks & Dracup, 2006; Perkins-Porras et al., 2009; Svensson et al., 2003).

Age, sex and marital status are variables which have always been under the influence of delay time in referring to hospital, although this study did not show a significant correlation among referring delay variables and these variables. Other various studies have stated aging in direct relation with referring delay time to hospital as an effective factor (Banks & Dracup, 2006; Gartner, Walz, Bauernschmitt, & Ladwig, 2008; Gharakhani, Naghsh Tabrizi, Emami, & Seif Rabiee, 2007; Goff et al., 2007; Khan et al., 2007; Leila Javadi, Masood Pezeshkian, Abbas Afrasiabi, Alireza Garjani, & Zahra Golmohammadi, 2010; Nguyen, Saczynski, Gore, & Goldberg, 2010; Perkins-Porras et al., 2009; Rezaey, Kohestany, Baghcheghy, & Yazdan Khah Fard, 2006; Sarı et al., 2008; Svensson et al., 2003; Taghadosi et al., 2007; Z, 2004). The results of a study in province of Sistan and Balouchestan (Iran) demonstrated that there was a significant relationship between hypertension, hyperlipidemia, diabetes mellitus, obesity and gender (Pishkarmofrad et al., 2012).

In some studies increase of referring delay time had been effective along aging (33-35). It was recognized in our study that referring delay time variables had been longer in women than men, which is similar to other studies (Nguyen et al., 2010; Perkins-Porras et al., 2009; Rezaey et al., 2006; Taghadosi et al., 2007). Patients’sex has not been related to referring delay time in some studies (Akbari et al., 2009; Gharakhani et al., 2007; Leila Javadi et al., 2010; Rezaey et al., 2006; Sarı et al., 2008; Svensson et al., 2003). The best effect of drug is at the first half of chest pain, highest mortalities also occur at first hours after MI and many factors have roles in creating them. The need for attending this issue, public training of society, coping with chest pain, recognizing risk factors and controlling them is felt more than past.
